# Mucopolysaccharidosis Type I in the Russian Federation and Other Republics of the Former Soviet Union: Molecular Genetic Analysis and Epidemiology

**DOI:** 10.3389/fmolb.2021.783644

**Published:** 2022-01-24

**Authors:** E. Yu Voskoboeva, T. M. Bookina, A. N. Semyachkina, S. V. Mikhaylova, N. D. Vashakmadze, G. V. Baydakova, E. Yu Zakharova, S. I. Kutsev

**Affiliations:** ^1^ Federal State Budgetary Scientific Institution, Research Center for Medical Genetics, Moscow, Russia; ^2^ Research and Clinical Institute of Pediatrics named after Yuri Veltischev, Pirogov Russian National Research Medical University, Ministry of Health of the Russian Federation, Moscow, Russia; ^3^ Detached Structural Unit Russian Children’s Clinical Hospital, Clinical Institute for Pediatrics, Pirogov Russian National Research Medical University, Ministry of Health of the Russian Federation Research, Moscow, Russia; ^4^ Pediatrics Department, Central Clinical Hospital of the Russian Academy of Sciences, Pirogov Russian National Research Medical University, Moscow, Russia

**Keywords:** mucopolysaccharidosis I, IDUA gene, genotype-phenotype, iduronidase, Russian Federation, Hurler, Hurler-Scheie, Scheie syndrome

## Abstract

Mutations in the *IDUA* gene cause deficiency of the lysosomal enzyme alpha-l-iduronidase (IDUA), which leads to a rare disease known as mucopolysaccharidosis type I. More than 300 pathogenic variants of the *IDUA* gene have been reported to date, but not much is known about the distribution of mutations in different populations and ethnic groups due to the low prevalence of the disease. This article presents the results of a molecular genetic study of 206 patients with mucopolysaccharidosis type I (MPS I) from the Russian Federation (RF) and other republics of the former Soviet Union. Among them, there were 173 Russian (Slavic) patients, 9 Tatars, and 24 patients of different nationalities from other republics of the former Soviet Union. Seventy-three different pathogenic variants in the *IDUA* gene were identified. The common variant NM_000203.5:c.208C>T was the most prevalent mutant allele among Russian and Tatar patients. The common variant NM_000203.5:c.1205G>A accounted for only 5.8% mutant alleles in Russian patients. Both mutations were very rare or absent in patients from other populations. The pathogenic variant NM_000203.5:c.187C>T was the major allele in patients of Turkic origin (Altaian, Uzbeks, and Kyrgyz). Specific own pathogenic alleles in the *IDUA* gene were identified in each of these ethnic groups. The identified features are important for understanding the molecular origin of the disease, predicting the risk of its development and creating optimal diagnostic and treatment tools for specific regions and ethnic groups.

## Background

MPS I is a rare lysosomal storage disease that results from the pathogenic nucleotide alterations in the *IDUA* gene. The *IDUA* gene encodes the lysosomal enzyme alpha-l-iduronidase (IDUA; EC 3.2.1.76) involved in glycosaminoglycan (GAG) metabolism. The IDUA deficiency leads to the accumulation of the two types of GAGs, i.e., heparan sulfate and dermatan sulfate in different tissues and organs, resulting in the development of progressive multisystem pathology (Campos and Monaga, 2012). The three subtypes of the disease are traditionally distinguished: severe form (Hurler syndrome; MPS IH; MIM#607014), intermediate form (Hurler/Scheie syndrome; MPS IH/S; MIM#607015), and mild form (Scheie syndrome MPS IS; MIM#607016). However, in patients with different MPS I syndromes, no easily measurable biochemical differences have been identified and the clinical findings overlap (Muenzer, 2004). It is now assessed that MPS I exists as a spectrum of disorders from the attenuated form to severe, with many phenotypes in between. The clinical symptoms include coarse face, growth retardation, corneal clouding, contractures of the joints, kyphoscoliosis, dysostosis multiplex, hearing loss, thickening of the heart valves, hepatosplenomegaly, diffuse muscle hypotension, umbilical and inguinal hernias, and cardiomyopathy. The manifestation and severity of symptoms vary depending on the severity of the disease. Cognitive and developmental delays are observed in patients with severe form of disease (Neufeld et al., 2001; Hampe et al., 2020).

The first step in diagnosing MPS I involves qualitative and quantitative analysis of urine GAGs and measurement of the residual alpha-l-iduronidase activity. Enzyme activity can be measured in plasma or leukocyte homogenate of patients, using phenyl-iduronide or 4-methylumbelliferyl as a substrate (Hall and Neufeld, 1973; Hopwood et al., 1979; Hopwood and Harrison, 1982; Stone, 1998). Since recently, enzyme activity has been measured in dried blood spots (DBS) by tandem mass spectrometry (MS/MS) (Kumar et al., 2015). The second step, which is considered definitive to confirm the disease, is the molecular genetic analysis of the *IDUA* gene.

The *IDUA* gene is located on the locus 4p16.3 of chromosome 4 and consists of 14 exons and 13 introns. The gene is transcribed into a 2.3-kb cDNA, which encodes a 653-residue glycopeptide (Scott et al., 1991; Scott et al., 1992). Three hundred nineteen variants in the *IDUA* gene have been reported in the Human Genetic Mutation Database (HGMD). Of these, 86 are nonsense and missense mutations, 49 are splicing substitutions, 47 are minor deletions, 23 are minor insertions, four are small indels, 10 are gross deletion, two are gross insertions, three are complex rearrangement, and one is regulatory substitution (data as of November 2021). Frequencies of mutations differ across populations (www.hgmd.cf.ac.uk – Human).

The most common pathogenic alleles worldwide are NM_000203.5:c.1205G>A and NM_000203.5:c.208C>T. The last investigation of the global distribution of common mutations in the *IDUA* gene has shown that the NM_000203.5:c.1205G>A was major allele among patients with MPS I from most European countries, America, and Australia. The common allele NM_000203.5:c.208C>T was found mostly in North and East Europe. The accumulation of unique pathogenic alleles is the characteristic of individual population groups. In different populations, the frequency of MPS I ranges from 0.11:100,000 to 1.85:100,000 newborns (Khan et al., 2017; Poletto et al., 2018).

Specific treatment options available for this disorder are Enzyme Replacement Therapy and allogeneic Hematopoietic Stem Cell Transplantation (Concolino et al., 2018; Kubaski et al., 2020).

Genotype–phenotype correlations in MPS I, as well as in other hereditary diseases, are not obvious. However, in some cases, a clear relationship between pathogenic variants and clinical manifestations can be traced (Clarke et al., 2019). Understanding genotype–phenotype correlations may be useful for clinical management and treatment decisions.

Currently, newborn screening for MPS I has been implemented, allowing for early identification of patients and timely treatment (Clarke et al., 2017). For screening to be effective, it is necessary to know the incidence of the disease in the population. In each population, the incidence of MPS I varies due to differences in ethnicity and/or founder effects. Besides, local ethnic groups still retain their unique gene pools.

Knowledge of the prevalence of MPS I and the identification of genetic characteristics of each ethnic group are the prerequisites for the development of optimal methods of diagnosis, treatment, and prediction of disease risk for specific regions and ethnic groups.

Of the 256 patients from different regions of Russia and the former Soviet Union diagnosed with MPS I in the last 35 years, DNA analysis was performed in 206 patients from 201 families.

The aim of the study was to perform a comprehensive DNA analysis of the *IDUA* gene, studying genotype–phenotype correlations and peculiarities of pathogenic variants among patients with MPS I from different ethnic groups.

## Materials and Methods

### Patients

A total of 256 patients (134 male and 122 female) were diagnosed with MPS I from 1985 through 2020. For 206 patients from 201 families, DNA samples were available, and the analysis of the *IDUA* gene was performed.

A group of Russian patients of Slavic origin was formed. According to the parents, both or at least one parent was Russian Slavic origin. The group included 173 patients from different regions of the RF. Other patients included Tatars (9), Armenians (6), Azerbaijanis (2), Kazakhs (3), Uzbeks (7), Altaian (1), Kyrgyz (1), Moldovans (1), Ukrainians (2), and Avars (1).

### Biochemical Methods

Electrophoresis of urinary GAGs was performed according to the standard method (Stone, 1998). Assay of alpha-l-iduronidase in peripheral blood leukocytes employed 0.01 M phenyl-iduronide, as previously described (Hall and Neufeld, 1973). Values were expressed as nanomoles of phenol liberated per milligram of protein in 18 h at ambient temperature. The value of the residual activity of IDUA in the range of 64–170 (nmol/18 h/mg) was considered normal.

From 2017, the activity of alpha-l-iduronidase has been measured in DBSs by MS/MS method (Chennamaneni et al., 2014). Alpha-l-iduronidase activity measurement was performed using a commercial kit according to the manufacturer’s manual.

### DNA Analysis

DNA was extracted following the manufacturer’s protocol with the DIAtomt DNA Prep100 kit (Isogene Lab. Ltd., Russia).

The 14 exons and exon–intron boundaries of the *IDUA* gene were amplified from DNA samples. Primers and PCR reaction conditions have been previously described (Beesley et al., 2001). Sanger sequencing of each one of the 14 exons was performed according to the manufacturer’s protocol on an ABI Prism 3500XL (Applied Biosystems). PCR products containing mutations were re-sequenced in both directions. The mutations were further confirmed where possible by restriction analysis (data not shown).

### Ethics Statements

Written informed consent was obtained from patients and their parents or legal guardians. Molecular research was approved by the ethics committee of the Federal State Budgetary Scientific Institution “Research Center for Medical Genetics” (Moscow, Russia). All procedures were in accordance with the ethical standards of the responsible committee on human experimentation (institutional and national) and with the Helsinki Declaration.

## Results

Patients were received initial consultations at regional medical genetic counseling clinics, the Scientific Clinical Institute of Pediatrics (Moscow), or the Russian Children’s Clinical Hospital (Moscow). Patients with suspected MPS I were referred for confirmation of the diagnosis to The Laboratory of Hereditary Metabolic Diseases of Federal State Budgetary Scientific Institution “Medical Genetics Research Center” (Moscow, Russia).

Electrophoresis of urine GAGs and measurement of lysosomal enzyme activity in peripheral blood leukocytes or DBSs were performed for all patients with suspected MPS I. Аll patients with MPS I had hyperexcretion of urine heparan and dermatan sulfate. Residual IdA activity in leukocytes varied from zero to 18.7 nmol/18 h/mg. Residual IdA activity in DBS was always below 0 μmol/h/L blood (the control values 1–7 μmol/h/L blood). No dependence of IdA activity on the severity of the clinical manifestation of the disease was observed (Table 1).

**TABLE 1:** Genotype and phenotype of patients with an indication of the place of residence and nationality.

Number of families; patients	Patients initials	IdA activity in leukocytes (nmol/18h/mg) or in DBS (μmol/h/L)	Genotype	Phenotype	Region of residence (federal district of RF or republics)
Russian Patients					
1	a.a	0,01 DBS	NM_000203.5:c.[208C>T];[208C>T]	Severe	NW
2	V.YA.	0,01 leukocytes	NM_000203.5:c.[208C>T];[208C>T]	Severe	NW
3	DR.VL.	0,01 leukocytes	NM_000203.5:c.[208C>T];[208C>T]	Severe	NW
4	ZDER.YA.	0,01 DBS	NM_000203.5:c.[208C>T];[208C>T]	Severe	NW
5	KOR.D.	0,01 leukocytes	NM_000203.5:c.[208C>T];[208C>T]	Severe	NW
6	KYD.K.	0,01 DBS	NM_000203.5:c.[208C>T];[208C>T]	Severe	NW
7	USH.A.	0,54 leukocytes	NM_000203.5:c.[208C>T];[208C>T]	Severe	NW
8	LUS.K.	0,01 DBS	NM_000203.5:c.[208C>T];[208C>T]	Severe	NW
9	MED.A.	4,5 leukocytes	NM_000203.5:c.[208C>T];[208C>T]	Severe	NW
10	P.V.	0,01 DBS	NM_000203.5:c.[208C>T];[208C>T]	Severe	NW
11;11a	S.E.	0,2 leukocytes	NM_000203.5:c.[208C>T];[208C>T]	Severe	NW
	S.A.	0,35 leukocytes	NM_000203.5:c.[208C>T];[208C>T]	Severe	NW
12	S.D.	0,01 DBS	NM_000203.5:c.[208C>T];[208C>T]	Severe	NW
13	C.Y.	0,01 DBS	NM_000203.5:c.[208C>T];[208C>T]	Severe	NW
14	CH.S.	0,01 leukocytes	NM_000203.5:c.[208C>T];[208C>T]	Severe	NW
15	BEL.A.	0,01 leukocytes	NM_000203.5:c.[208C>T];[208C>T]	Severe	C
16	VOR.K.	0,01 DBS	NM_000203.5:c.[208C>T];[208C>T]	Severe	C
17	VOR.M.	2,15 leukocytes	NM_000203.5:c.[208C>T];[208C>T]	Severe	C
18	GAI.S.	0,01 leukocytes	NM_000203.5:c.[208C>T];[208C>T]	Severe	C
19	DAR.A.	0,01 leukocytes	NM_000203.5:c.[208C>T];[208C>T]	Severe	C
20	IV.S.	0,01 DBS	NM_000203.5:c.[208C>T];[208C>T]	Severe	C
21	KAR.R.	0,01 DBS	NM_000203.5:c.[208C>T];[208C>T]	Severe	C
22	KL.L.	0,01 leukocytes	NM_000203.5:c.[208C>T];[208C>T]	Severe	C
23	KOZ.A.	4,4 leukocytes	NM_000203.5:c.[208C>T];[208C>T]	Severe	C
24	KR.M.	0,01 leukocytes	NM_000203.5:c.[208C>T];[208C>T]	Severe	C
25	KYZ.M.	0,01 leukocytes	NM_000203.5:c.[208C>T];[208C>T]	Severe	C
26	KYZ.V.	0,01 leukocytes	NM_000203.5:c.[208C>T];[208C>T]	Severe	C
27	LEON.M.	1,4 leukocytes	NM_000203.5:c.[208C>T];[208C>T]	Severe	C
28	ROM.I.	0,01 leukocytes	NM_000203.5:c.[208C>T];[208C>T]	Severe	C
29	POM.A.	1,1 leukocytes	NM_000203.5:c.[208C>T];[208C>T]	Severe	C
30	PL.V.	0,01 leukocytes	NM_000203.5:c.[208C>T];[208C>T]	Severe	C
31	POP.V.	0,01 DBS	NM_000203.5:c.[208C>T];[208C>T]	Severe	C
32	SOP.M.	4,4 leukocytes	NM_000203.5:c.[208C>T];[208C>T]	Severe	C
33	SP.A.	0,01 leukocytes	NM_000203.5:c.[208C>T];[208C>T]	Severe	C
34	S.DM.	0,01 leukocytes	NM_000203.5:c.[208C>T];[208C>T]	Severe	C
35	887	0,01 DBS	NM_000203.5:c.[208C>T];[208C>T]	Severe	C
36	SH.M.	3,5 leukocytes	NM_000203.5:c.[208C>T];[208C>T]	Severe	P
37	YAK.E.	0,01 leukocytes	NM_000203.5:c.[208C>T];[208C>T]	Severe	P
38	ANT.E.	0,01 DBS	NM_000203.5:c.[208C>T];[208C>T]	Severe	P
39	NIK.M.	5,2 leukocytes	NM_000203.5:c.[208C>T];[208C>T]	Severe	P
40	RAV.F.	0,01 leukocytes	NM_000203.5:c.[208C>T];[208C>T]	Severe	P
41	UCH.A.	0,01 leukocytes	NM_000203.5:c.[208C>T];[208C>T]	Severe	P
42	GOL.M.	0,01 leukocytes	NM_000203.5:c.[208C>T];[208C>T]	Severe	P
43	JEL.K.	0,01 DBS	NM_000203.5:c.[208C>T];[208C>T]	Severe	P
44	JYR.M.	0,01 DBS	NM_000203.5:c.[208C>T];[208C>T]	Severe	P
45	KAR.K.	0,01 leukocytes	NM_000203.5:c.[208C>T];[208C>T]	Severe	P
46	AN.K.	0,01 DBS	NM_000203.5:c.[208C>T];[208C>T]	Severe	P
47	DAV.E.	0,01 DBS	NM_000203.5:c.[208C>T];[208C>T]	Severe	STH
48	DR.A.	0,01 leukocytes	NM_000203.5:c.[208C>T];[208C>T]	Severe	STH
49	JUR.A.	0,01 leukocytes	NM_000203.5:c.[208C>T];[208C>T]	Severe	STH
50	KYZ.A.	0,2 leukocytes	NM_000203.5:c.[208C>T];[208C>T]	Severe	U
51	BOR.M.	0,01 leukocytes	NM_000203.5:c.[208C>T];[208C>T]	Severe	U
52	ROM.T.	0,01 DBS	NM_000203.5:c.[208C>T];[208C>T]	Severe	U
53	AL.M.	2,1 leukocytes	NM_000203.5:c.[208C>T];[208C>T]	Severe	S
54	KYR.A.	0,01 leukocytes	NM_000203.5:c.[208C>T];[208C>T]	Severe	S
55	KIR.N.	8,4 leukocytes	NM_000203.5:c.[208C>T];[208C>T]	Severe	S
56	GER.N.	1,7 leukocytes	NM_000203.5:c.[208C>T];[208C>T]	Severe	S
57	MIX.EV.	3,8 leukocytes	NM_000203.5:c.[208C>T];[208C>T]	Severe	S
58	P.M.	0,01 DBS	NM_000203.5:c.[208C>T];[208C>T]	Severe	S
59	B.K.	0,01 DBS	NM_000203.5:c.[208C>T];[208C>T]	Severe	S
60	S.S.	0,01 DBS	NM_000203.5:c.[208C>T];[208C>T]	Severe	E
61	OR.V.	2,3 leukocytes	NM_000203.5:c.[208C>T];[208C>T]	Severe	E
62	OB.D.	2,2 leukocytes	NM_000203.5:c.[208C>T];[208C>T]	Severe	E
63	KOR.ST	8,5 leukocytes	NM_000203.5:c.[208C>T];[208C>T]	Severe	E
64	TIK.M.	0,01 leukocytes	NM_000203.5:c.[208C>T];[1205G>A]	Severe	NW
65	ER.E.	0,01 leukocytes	NM_000203.5:c.[208C>T];[1205G>A]	Severe	C
66	PL.AN	0,01 leukocytes	NM_000203.5:c.[208C>T];[1205G>A]	Severe	C
67	R.	0,01 leukocytes	NM_000203.5:c.[208C>T];[1205G>A]	Severe	C
68	SH.S.	3,2 leukocytes	NM_000203.5:c.[208C>T];[1205G>A]	Severe	C
69	KIR.S.	0,01 leukocytes	NM_000203.5:c.[208C>T];[1205G>A]	Severe	STH
70; 70a	K.D	0,8 leukocytes	NM_000203.5:c.[208C>T];[1205G>A]	Severe	U
	K.S	3,7 leukocytes	NM_000203.5:c.[208C>T];[1205G>A]	Severe	U
71	L.SER.	0,01 DBS	NM_000203.5:c.[208C>T];[1205G>A]	Severe	u
72	FOM.L.	0,01 DBS	NM_000203.5:c.[208C>T];[1205G>A]	Severe	S
73	KAB.E.	0,01 DBS	NM_000203.5:c.[208C>T];[1205G>A]	Severe	S
74	JYR.O.	0,01 leukocytes	NM_000203.5:c.[1205G>A];[1205G>A]	Severe	STH
75	BAT.E.	0,01 leukocytes	NM_000203.5:c.[1205G>A]; [1688A>C]	Attenuated	NW
76	ZOT.YU.	0,01 leukocytes	NM_000203.5:c.[1139A>G];[1205G>A]	Attenuated	C
77	PL.A.	1,2 leukocytes	NM_000203.5:c.[1139A>G];[1205G>A]	Attenuated	C
78	PROM.E.	0,01 leukocytes	NM_000203.5:c.[1139A>G];[1205G>A]	Attenuated	C
79	SH.A.	0,01 DBS	NM_000203.5:c.[1205G>A]; [1898C>A]	Severe	C
80	OR.A.	0,01 DBS	NM_000203.5:c.[1205G>A]; [1898C>A]	Severe	C
81	KOP.N.	0,01 leukocytes	NM_000203.5:c.[1205G>A];[1873_1888delinsACA]	Severe	C
82	BON.E.	3,5 leukocytes	NM_000203.5:c.[208C>T];[1139A>G]	Attenuated (MPS IS)	C
83	GR.V.	2,85 leukocytes	NM_000203.5:c.[208C>T];[1139A>G]	Attenuated	C
84	IS.M.	0,01 leukocytes	NM_000203.5:c.[208C>T];[1139A>G]	Attenuated (MPS IS)	C
85	M.AL	2,8 leukocytes	NM_000203.5:c.[208C>T];[1139A>G]	Attenuated	C
86	MIW.E.	1,42 leukocytes	NM_000203.5:c.[208C>T];[1139A>G]	Attenuated	C
87	KYL.O.	3,8 leukocytes	NM_000203.5:c.[208C>T];[1139A>G]	Attenuated	STH
88	SH.M.	0,01 DBS	NM_000203.5:c.[208C>T];[1139A>G]	Attenuated	STH
89	HM.A.	0,01 DBS	NM_000203.5:c.[208C>T];[1139A>G]	Attenuated	S
90	BAR.E.	0,01 leukocytes	NM_000203.5:c.[208C>T];[1139A>G]	Attenuated	S
91; 90a	SH.DM	0,01 DBS	NM_000203.5:c.[1139A>G];[1139A>G]	Attenuated (MPS IS)	C
	SH.YU	0,01 DBS	NM_000203.5:c.[1139A>G];[1139A>G]	Attenuated (MPS IS)	C
92	VL.D	0.5 leukocytes	NM_000203.5:c.[1139A>G];[1676T>C]	Attenuated (MPS IS)	C
93	KL.M.	0,01 DBS	NM_000203.5:c.[967_969del];[1139A>G]	Attenuated	C
94	MIL.A.	0,1 leukocytes	NM_000203.5:c.[1139A>G];[1873_1888delinsACA]	Attenuated	U
95	ZAN.K.	0,05 leukocytes	NM_000203.5:c.[208C>T];[1115A>G]	Attenuated	C
96	SIV.A.	18,7 leukocytes	NM_000203.5:c.[208C>T];[1115A>G]	Attenuated	C
97	SH.K.	2,4 leukocytes	NM_000203.5:c.[208C>T];[1115A>G]	Attenuated	STH
98	HM.S.	2,4 leukocytes	NM_000203.5:c.[208C>T];[1115A>G]	Attenuated (MPS IS)	STH
99; 99a	B.O.	2,1 leukocytes	NM_000203.5:c.[1115A>G];[1115A>G]	Attenuated (MPS IS)	STH
	B.D	0,1 leukocytes	NM_000203.5:c.[1115A>G];[1115A>G]	Attenuated (MPS IS)	STH
100	POL.E	0,01 leukocytes	NM_000203.5:c.[1115A>G];[1688A>C]	Attenuated (MPS IS)	C
101	KOL.P.	0,01 leukocytes	NM_000203.5:c.[208C>T];[1598C>T]	Severe	E
102	KOR.S.	2,5 leukocytes	NM_000203.5:c.[208C>T];[1598C>T]	Severe	STH
103	BAB.V.	0,01 DBS	NM_000203.5:c.[208C>T];[1598C>T]	Severe	STH
104	F.A.	0,01 DBS	NM_000203.5:c.[208C>T];[1598C>T]	Severe	C
105	BOL.M.	5,0 leukocytes	NM_000203.5:c.[878_889dup];[1598C>T]	Attenuated (MPS IS)	C
106	BUI.K.	2,7 leukocytes	NM_000203.5:c.[123G>A];[208C>T]	Severe	P
107	P.D.	1,73 leukocytes	NM_000203.5:c.[140G>A];[208C>T]	Severe	NW
108	MOR.V.	0,01 DBS	NM_000203.5:c.[208C>T];[1029C>A]	Severe	C
109	PR.P.	0,01 leukocytes	NM_000203.5:c.[208C>T];[1029C>A]	Severe	E
110	TAR.P.	14,1 leukocytes	NM_000203.5:c.[208C>T];[1219C>T]	Severe	c
111	IV.A.	0,01 leukocytes	NM_000203.5:c.[208C>T];[1855C>T]	Severe	E
112	AN.P.	5,9 leukocytes	NM_000203.5:c.[208C>T];[1861C>G]	Severe	STH
113	KICH.YA.	2,2 leukocytes	NM_000203.5:c.[208C>T];[1898C>A]	Severe	E
114	P.K.	0,01 leukocytes	NM_000203.5:c.[208C>T];[1898C>A]	Severe	U descendant of a mixed marriage Russian/Armenian
115	SID.A	0,01 leukocytes	NM_000203.5:c.[208C>T];[1898C>A]	Severe	U descendant of a mixed marriage Russian/Turkmen
116	DUR.A.	2,0 leukocytes	NM_000203.5:c.[1A>C];[208C>T]	Severe	C
117	KOL.SV	0,01 leukocytes	NM_000203.5:c.[208C>T];[223G>C]	Severe	C
118	M.S.	0,01 leukocytes	NM_000203.5:c.[208C>T];[223G>A]	Severe	NW
119	SM.B.	0,01 leukocytes	NM_000203.5:c.[208C>T];[223G>A]	Severe	C
120	SAM.I.	0,01 leukocytes	NM_000203.5:c.[208C>T];[266G>A]	Attenuated	P
121	BUR.A.	0,01 leukocytes	NM_000203.5:c.[208C>T];[531C>G]	Attenuated	C
122	SYH.E.	2,2 leukocytes	NM_000203.5:c.[208C>T];[589G>A]	Attenuated	C
123	MAM.	0,01 DBS	NM_000203.5:c.[208C>T];[793G>C]	Attenuated	C
124	RAS.I.	0,01 leukocytes	NM_000203.5:c.[208C>T];[826G>A]	Attenuated	STH
125	V.AL.	3,4 leukocytes	NM_000203.5:c.[208C>T];[979G>C]	Severe	C
126	SEL.D.	2,16 leukocytes	NM_000203.5:c.[208C>T];[1150A>G]	Severe	P
127	VIL.D.	11,7 leukocytes	NM_000203.5:c.[208C>T];[1321T>A]	Attenuated	P
128	GOR.E.	0,01 leukocytes	NM_000203.5:c.[208C>T];[1459T>C]	Severe	P
129	NIK.K.	6,1 leukocytes	NM_000203.5:c.[208C>T];[1475G>C]	Attenuated	P
130	YAK.A.	0,01 DBS	NM_000203.5:c.[208C>T];[1505G>C]	Attenuated	P
131	V.EL.	0,01 leukocytes	NM_000203.5:c.[208C>T];[1513C>G]	Severe	C descendant of a mixed marriage Russian/Azerbaijanian
132	L.M.	0,01 DBS	NM_000203.5:c.[208C>T];[1600T>C]	Attenuated	C
133	P.A.	0,01 leukocytes	NM_000203.5:c.[208C>T];[1622G>T]	Attenuated	C
134	GL.E.	3,1 leukocytes	NM_000203.5:c.[208C>T];[1664G>C]	Attenuated	C
135	CH.V.	1,28 leukocytes	NM_000203.5:c.[208C>T];[1688A>C]	Attenuated	P
136	SH.N.	6,4 leukocytes	NM_000203.5:c.[208C>T];[1688A>C]	Severe	STH
137	MED.A.	0,1 leukocytes	NM_000203.5:c.[208C>T];[1898C>T]	Attenuated	E
138	KYL.V	0,01 DBS	NM_000203.5:c.[35_46del];[208C>T]	Severe	S
139	AHM.S.	5,6 leukocytes	NM_000203.5:c.[208C>T];[222_226del]	Severe	NW
140	C.A.	0,01 DBS	NM_000203.5:c.[208C>T];[584_589+8del]	Severe	C
141	LI.M	0,01 leukocytes	NM_000203.5:c.[208C>T];[683del]	Severe	NW descendant of a mixed marriage Russian/Korean
142	SV.I.	0,01 leukocytes	NM_000203.5:c.[208C>T];[705_707del]	Severe	C
143	BR.A	0,01 leukocytes	NM_000203.5:c.[208C>T];[923_932del]	Severe	U
144	G.D.	4,5 leukocytes	NM_000203.5:c.[208C>T];[1045_1047del]	Severe	U
145	PL.E.	4,45 leukocytes	NM_000203.5:c.[208C>T];[1238_1264del]	Severe	NW
146	KIR.M.	0,01 leukocytes	NM_000203.5:c.[208C>T];[1238_1264del]	Severe	U
147	PROP.I.	0,01 leukocytes	NM_000203.5:c.[208C>T];[1238_1264del]	Severe	P
148; 148a	R.K.	14,8 leukocytes	NM_000203.5:c.[1459T>C];[1238_1264del]	Severe	P
	R.KR.	11,4 leukocytes	NM_000203.5:c.[1459T>C];[1238_1264del]	Severe	P
149	AN.T.	8,7 leukocytes	NM_000203.5:c.[208T>C];[1614del]	Severe	C
150	KAG.R.	0,01 leukocytes	NM_000203.5:c.[208T>C];[1847del]	Severe	U
151	PON.YU.	0,1 leukocytes	NM_000203.5:c.[208T>C];[811_816dup]	Severe	U
152	ZAR.P.	4,5 leukocytes	NM_000203.5:c.[208T>C];[878_889dup]	Attenuated	C
153	KR.S.	0,01 leukocytes	NM_000203.5:c.[208T>C];[1092dup]	Severe	P
154	GR.V.	1,09 leukocytes	NM_000203.5:c.[208T>C];[1742dup]	Severe	NW
155	YAN.K.	0,01 DBS	NM_000203.5:c.[208T>C];[1781dup]	Severe	P
156	TOR.R.	2,7 leukocytes	NM_000203.5:c.[1873_1888delinsACA];[1873_1888delinsACA]	Attenuated	STH
157	NIF.	3,6 leukocytes	NM_000203.5:c.[208C>T];[1873_1888delinsACA]	Attenuated	STH
158	ARS.A.	0,01 DBS 12,1 leukocytes	NM_000203.5:c.[46_57del];[1873_1888delinsACA]	Severe	STH
159	KAR.S.	0,01 leukocytes	NM_000203.5:c.[208C>T];[1403-3C>G]	Severe	C
160	LEON.V.	0,01 leukocytes	NM_000203.5:c.[208C>T];[1524+1G>A]	Severe	P
161	KAL.M.	0,01 leukocytes	NM_000203.5:c.[208C>T];[1650+5G>A]	Severe	STH
162	VOD.K.	0,01 DBS	NM_000203.5:c.[208C>T];[1650+5G>A]	Severe	U
163	ZIK.YA.	0,01 leukocytes	NM_000203.5:c.[208C>T];[1650+5G>A]	Severe	S
164	P.L.	0,01 DBS	NM_000203.5:c.[1650+5G>A];[1650+5G>A]	Severe	S
165	BUD.A.	0,4 leukocytes	NM_000203.5:c.[718C>G];[ 1044C>G]	Attenuated	C
166	S.DM.	0,01 DBS	NM_000203.5:c.[1601C>A];[ 1743C>G]	Severe	P
167	K.E.	0,01 leukocytes	NM_000203.5:c.[208C>T];[?]	Attenuated	C
168	GAI.G.	0,01 DBS	NM_000203.5:c.[1205G>A];[?]	Severe	STH
Patients of other nationalities					
169	T.V.	0,01 DBS	NM_000203.5:c[208C>T];[208C>T]	Severe	Tatar/Tatarstan
170	MYX.A.	0,01 leukocytes	NM_000203.5:c[208C>T];[208C>T]	Severe	Tatar/Tatarstan
171	MYX.I	0,01 leukocytes	NM_000203.5:c[208C>T];[208C>T]	Severe	Tatar/Tatarstan
172	A.AN.	0,01 leukocytes	NM_000203.5:c[208C>T];[1688A>C]	Severe	Tatar/Tatarstan
173	GR.K	5,7 leukocytes	NM_000203.5:c[208C>T];[1037T>G]	Attenuated	Tatar/Tatarstan
174	S.ID.	0,01 leukocytes	NM_000203.5:c[208C>T];[1166C>A]	Attenuated	Tatar/Tatarstan
175	AB.S.	0,01 leukocytes	NM_000203.5:c[208C>T];[1099_1007delinsAGGTCAC]	Severe	Tatar/Tatarstan
176	MYL.B.	0,01 leukocytes	NM_000203.5:c.[46_57del];[1139A>G]	Attenuated	Tatar/Tatarstan
177	ABD.S.	0,01 DBS	NM_000203.5:c.[1139A>G];[?]	Attenuated	Tatar/Tatarstan
178	KYI.E.	5,4 leukocytes	NM_000203.5:c.[187C>T];[187C>T]	Severe	Altaian/Altai Republic
179	D.A.	0,01 DBS	NM_000203.5:c.[187C>T];[187C>T]	Severe	Uzbek/Uzbekistan
180	AT.H.	0,01 DBS	NM_000203.5:c.[187C>T];[187C>T]	Severe	Uzbek/Uzbekistan
181	MOM.I.	0,01 DBS	NM_000203.5:c.[187C>T];[187C>T]	Severe	Uzbek/Uzbekistan
182	MM	0,01 DBS	NM_000203.5:c.[187C>T];[187C>T]	Severe	Uzbek/Uzbekistan
183	NISH.I	2,5 leukocytes	NM_000203.5:c.[187C>T];[187C>T]	Severe	Uzbek/Uzbekistan
184	I.OL.	0,01 leukocytes	NM_000203.5:c.[1882C>T];[1882C>T]	Severe	Uzbek/Uzbekistan
185	H.A.	0,01 leukocytes	NM_000203.5:c.[187C>T];[1030C>G]	Severe	Uzbek/Uzbekistan
186	K.N.	0,01 DBS	NM_000203.5:c.[1A>C];[187C>T]	Severe	Kyrgyz/Kyrgyzstan
187	NOV.O.	5,5 leukocytes	NM_000203.5:c.[250G>C];[250G>C]	Severe	Azerbaijani/Azerbaijan
188	AL.EM	0,01 leukocytes	NM_000203.5:c.[1A>C];[1A>C]	Severe	Azerbaijani/Azerbaijan
189	ARSH.K.	0,01 leukocytes	NM_000203.5:c.[1A>C];[1A>C]	Severe	Armenian/Armenia
190	P.S.	0,01 DBS	NM_000203.5:c.[1A>C];[510delinsAAGTTCCA]	Severe	Armenian/Armenia
191	SIM.E.	4,2 leukocytes	NM_000203.5:c.[1A>C];[510delinsAAGTTCCA]	Severe	Armenian/Armenia
192	VAS.E.	0,01 leukocytes	NM_000203.5:c.[510delinsAAGTTCCA];[ 1049A>G]	attenuated	Armenian/Armenia
193	S.	0,01 leukocytes	NM_000203.5:c.[510delinsAAGTTCCA];[510delinsAAGTTCCA]	Severe	Armenian/Armenia
194	G.G.	0,01 leukocytes	NM_000203.5:c.[1898C>A];[1898C>A]	Severe	Armenian/Armenia
195	B.A.	0,01 DBS	NM_000203.5:c.[1403-1g>t];[1403-1g>t]	Severe	Kazakh/Kazakhstan
196	B.S.	0,01 leukocytes	NM_000203.5:c.[1205G>A];[1403-1g>t]	Severe	Kazakh/Kazakhstan
197	SYL.G.	0,01 leukocytes	NM_000203.5:c.[1403-1g>t];[1451_1480del]	Severe	Kazakh/Kazakhstan
198	K.D.	0,01 leukocytes	NM_000203.5:c.[208C>T];[0.208C>T]	Severe	Ukrainian/Ukraine
199	K.N.	9,1 leukocytes	NM_000203.5:c.[208C>T];[972+2T>C]	Severe	Ukrainian/Ukraine
200	AH.AH.	0,01 leukocytes	NM_000203.5:c.[166del];[166del]	Severe	Avar/Dagestan
201	G.AR.	0,01 DBS	NM_000203.5:c.[653C>T];[1398del]	Severe	Moldovan/Moldova

Federal districts of the Russian Federation: C, central; NW, Northwest; STH, South; P, Privolzhsky; U, Ural; S, Siberia; E, Far East.

### DNA Analysis

As a result of DNA sequencing analysis, 73 special mutations in different combination were revealed. Of them, 14 were nonsense mutations, 31 were missense mutations, 15 were small deletion, five were small insertions, three were small insdel, and five were site-splicing mutations. Forty-one mutations were well known or previously described. Thirty-two mutant alleles were not described before, and data on these nucleotide substitutions are not available in the HGMD or ClinVar databases (Table 2).

**TABLE 2:** Characteristics of the nucleotide variants detected in the *IDUA* gene.

*n*/n	Nucleotide variant	HGMD database accession	ClinVar database accession	Allele amount	Comment	References
	Protein variant					
	The *IDUA* gene exon					
NONSENSE MUTATIONS						
1	NM_000203.5:c.123G>A	CM113971	Not reported	1	Described in a Chinese patient with MPS IH	Wang et al. (2012)
	NP_000194.2:p.Trp41Ter					
	Exon 1					
2	NM_000203.5:c.140G>A	n.d.	Not reported	1	This study	—
	NP_000194.2:p.Trp47Ter					
	Exon1					
3	NM_000203.5:c.187C>T	CM981060	Not reported	14	Described by the authors in a patient from Uzbekistan	Voskoboeva et al. (1998)
	NP_000194.2:p.Gln63Ter					
	Exon 2					
4	NM_000203.5:c.208C>T	CM930424	VCV000011909.26 pathogenic	225	second common allele worldwide	Clarke and Scott (1993; Poletto et al. (2018)
	NP_000194.2:p.Gln70Ter					
	Exon 2					
5	NM_000203.5:c.1029C>A	CM981062	VCV000222997 pathogenic	2	Described by the authors in a patient from Uzbekistan	Voskoboeva et al. (1998)
	NP_000194.2:p.Tyr343Ter					
	Exon 8					
6	NM_000203.5:c.1029C>G	CM940972	VCV000550474 pathogenic/likely pathogenic	1	Described in Chinese and Iranian patients with MPS IH. This change creates a premature stop codon. RNA analysis indicates that this variant induces altered splicing and likely results in the loss of 19 amino acid residues but is expected to preserve the integrity of the reading-frame	Tieu and Menon (1994), Lee-Chen and Wang (1997), Kamranjam and Alaei (2019)
	NP_000194.2:p.Tyr343Ter					
	Exon 8					
7	NM_000203.5:c.1205G>A	CM920372	VCV000011908.26 pathogenic	22	First common allele worldwide	Clarke and Scott (1993; Poletto et al. (2018)
	NP_000194.2:p.Trp402Ter					
	Exon 9					
8	NM_000203.5:c.1219C>T	n.d.	VCV000983616.1 likely pathogenic	1	This study	---
	NP_000194.2:p.Gln407Ter					
	Exon 9					
					The variant was assessed in the context of healthy population screening (ClinVar)	
9	NM_000203.5: c.1601C>A	CM046175	n.d.	1	Described in Korean patient with MPS IH	Lee et al. (2004)
	NP_000194.2:p.Ser534Ter					
	Exon 11					
10	NM_000203.5: c.1743C>G	CM113562	VCV000550883.5 pathogenic	1	Described in European and Algerian patients with MPS IH	Bertola et al. (2011), Tebani et al. (2016)
	NP_000194.2:p.Tyr581Ter					
	Exon 13					
					The change creates a premature translational stop signal. It is expected to result in an absent or disrupted protein product	
11	NM_000203.5:c.1855C>T	CM013755	VCV000280976 pathogenic	1	Described in at least 12 (MPS IH or MPS IH/S) individuals	Beesley et al. (2001), Trofimova (2016), Uttarilli et al. (2016)
	NP_000194.2:p.Arg619Ter					
	Exon 14					
					The change results in a premature stop codon. While this is not anticipated to result in nonsense mediated decay, it is expected to disrupt the last 35 amino acids of the IDUA protein	
12	NM_000203.5:c.1861C>T	CM940974	VCV000011917 pathogenic	1	Described in several patients with MPS I	Bertola et al. (2011), Trofimova (2016), Uttarilli et al. (2016), Ghosh et al. (2017)
	NP_000194.2:p.Arg621Ter					
	Exon 14					
					The change results in a premature translational stop signal	
					While this is not anticipated to result in nonsense mediated decay, it is expected to disrupt the last 33 amino acids of the IDUA protein.	
13	NM_000203.5:c.1882C>T	CM013756	VCV000550421.4 pathogenic	2	Described in several patients with MPS I	Beesley et al. (2001), Bertola et al. (2011), Tebani et al. (2016), Uttarilli et al. (2016)
	NP_000194.2:p.Arg628Ter					
	Exon 14					
					The change results in a premature termination codon, predicted to cause a truncation of the encoded protein or absence of the protein due to nonsense mediated decay	
14	NM_000203.5: c.1898C>A	n.d.	not reported	7	Described in Ukrainian patients with MPS IH/S	Trofimova (2016)
	NP_000194.2:p.Ser633Ter					
	Exon 14					
MISSENSE MUTATIONS						
15	NM_000203.5:c.1A>C	CM970760	VCV000550458.2 pathogenic	8	Described in patients with MPS IH	Bertola et al. (2011), Atçeken et al. (2016), Shafaat et al. (2019)
	NP_000194.2:p.Met1Leu					
	Exon 1					
					The change affects the initiator methionine of the IDUA mRNA	
					The next in-frame methionine is located at codon 133	
					Most common in Iranian patient**s**	
16	NM_000203.5:c.223G>A	CM940969	VCV000222993.8 pathogenic	2	Described in patients with MPS IH	Beesley et al. (2001), Ghosh et al. (2017), Chkioua et al. (2018)
	NP_000194.2:p.Ala75Thr					
	Exon 2					
17	NM_000203.5:c.223G>C	CM981061	Not reported	1	Described by the authors in a patient from Russia	Voskoboeva et al. (1998)
	NP_000194.2:p.Ala75Pro					
	Exon 2					
18	NM_000203.5:c.250G>A	CM113552	VCV000726495.5 llikely pathogenic	2	Described in an Iranian patient with MPS I	Taghikhani et al. (2019)
	NP_000194.2:p.Gly84Ser					
	Exon 2					
19	NM_000203.5:c.266G>A	CM950677	VCV000011922.5 pathogenic	1	This variant in IDUA has been reported in 13 MPS I individuals with attenuated form	Yamagishi et al. (1996), Hein et al. (2003), Wang et al. (2012)
	NP_000194.2:p.Arg89Gln					
	Exon 2					
20	NM_000203.5:c.531C>G	n.d.	Not reported	1	This study	—
	NP_000194.2:p.Phe177Leu					
	Exon 5					
21	NM_000203.5:c.589G>A	CM113248	Not reported	1	Described in a patient with MPS I	Ghosh et al. (2017)
	NP_000194.2:p.Gly197Ser					
	Exon 5					
22	NM_000203.5:c.653T>C	CM940970	VCV000222995.3 pathogenic	1	Described in patients with MPS IH	Pollard et al. (2013)
	NP_000194.2:p.Leu218Pro					
	Exon 6					
23	NM_000203.5:c.718C>G	CM146929	Not reported	1	Described in a patient with MPS I	Chistiakov et al. (2014)
	NP_000194.2:p.His240Asn					
	Exon 6					
24	NM_000203.5:c.793G>C	CM042364	VCV000638074.3 pathogenic/likely pathogenic	1	Described in patients with MPS IH/S or MPS IS	Bertola et al. (2011); Clarke et al. (2019)
	NP_000194.2:p.Gly265Arg					
	Exon 6					
25	NM_000203.5:c.826G>A	CM110991	Not reported	1	Described in Thai patients with MPS IS and MPS IH/S	Prommajan et al. (2011)
	NP_000194.2:p.Glu276Lys					
	Exon 7					
26	NM_000203.5:c.979G>C	CM950680	VCV000167190.16 pathogenic	1	Described in patients with MPS IH	Bunge et al. (1995), Zanetti et al. (2019)
	NP_000194.2:p.Ala327Pro					
	Exon 8					
27	NM_000203.5:c.1037T>G	CM000404	VCV000011927.5 pathogenic	1	Described in patients with MPS IH/S	Teng et al. (2000), Lee et al. (2004), Wang et al. (2012)
	NP_000194.2:p.Leu346Arg					
	Exon 8					
					Common cause of disease in East	
					Asian population	
28	NM_000203.5:c.1044C>G	CM113557	VCV000557870.2 pathogenic/likely pathogenic	1	Described in patients with MPS IS	Bertola et al. (2011)
	NP_000194.2:p.Asn348Lys					
	Exon 8					
29	NM_000203.5:c.1049A>G	CM034102	VCV000635306.1 uncertain significance	1	Described in patients with MPS IS	Matte et al. (2003)
	NP_000194.2:p.Asn350Ser					
	Exon 8					
30	NM_000203.5:c.1115A>G	CM1614957	VCV000554765.1 uncertain significance	9	Described in patients with MPS IH	Trofimova (2016), Uttarilli et al. (2016)
	NP_000194.2:p.Asn372Ser					
	Exon 8					
31	NM_000203.5:c.1139A>G	CM950682	VCV000550799.1 pathogenic	21	Described in patients with MPS I	Scott et al. (1995), Venturi et al. (2002), Matte et al. (2003), Vazna et al. (2009)
	NP_000194.2:p.Gln380Arg					
	Exon 8					
32	NM_000203.5:c.1150A>G	n.d.	Not reported	1	This study	—
	NP_000194.2:p.Lys384Asn					
	Exon 8					
33	NM_000203.5:c.1166C>A	n.d.	Not reported	1	This study	—
	NP_000194.2:p.Ala389Asp					
	Exon 8					
34	NM_000203.5:c.1321T>A	n.d.	Not reported	1	This study	—
	NP_000194.2:p.Tyr441Asn					
	Exon 9					
35	NM_000203.5:c.1459T>C	n.d.	Not reported	3	This study	—
	NP_000194.2:p.Trp487Arg					
	Exon 10					
36	NM_000203.5:c.1475G>C	CM950686	VCV000011918.1 pathogenic/likely pathogenic	1	Described in a patient with MPS IS	Tieu et al. (1995)
	NP_000194.2:p.Arg492Pro					
	Exon 10					
37	NM_000203.5:c.1505G>C	n.d.	Not reported	1	This study	—
	NP_000194.2:p.Arg502Pro					
	Exon 10					
38	NM_000203.5:c.1513C>G	n.d.	Not reported	1	This study	—
	NP_000194.2:p.Arg505Gly					
	Exon 10					
39	NM_000203.5:c.1598C>T	CM981063	VCV000429205.3 likely pathogenic	5	Described in patients with MPS I	Voskoboeva et al. (1998), Atçeken et al. (2016)
	NP_000194.2:p.Pro533Leu					
	Exon 11					
40	NM_000203.5:c.1600T>C	n.d.	Not reported	1	This study	
	NP_000194.2:p.Ser534Pro					
	Exon 11					—
41	NM_000203.5:c.1622G>T	n.d.	Not reported	1	This study	—
	NP_000194.2:p.Cys541Phe					
	Exon 11					
42	NM_000203.5:c.1664G>C	n.d.	Not reported	1	This study	—
	NP_000194.2:p.Arg555Pro					
	Exon 12					
43	NM_000203.5:c.1676T>C	n.d.	Not reported	1	This study	—
	NP_000194.2:p.Leu559Pro					
	Exon 12					
44	NM_000203.5:c.1688A>C	n.d.	Not reported	5	Described in Ukrainian patients with MPS IH/S	Trofimova (2016)
	NP_000194.2:p.Gln563Pro					
	Exon 12					
45	NM_000203.5:c.1898C>T	CM013757	VCV000556406	1	Described in patients with MPS I	Beesley et al. (2001), Wang et al. (2012), Uttarilli et al. (2016)
	NP_000194.2:p.Ser633Leu					
	Exon 14					
SMALL DELETIONS						
46	NM_000203.5:c.35_46del	n.d.	n.d.	1	Described in a patient with MPS I	Venturi et al. (2002)
	NP_000194.2:p.Leu13_Ser16del					
	Exon 1					
					Signal protein	
47	NM_000203.5:c.46_57del	CD941709	n.d.	2	Described in patients with MPS I	Bunge et al. (1994)
	NP_000194.2:p.Ser16_Ala19del					
	Exon1					
					Signal protein	
48	NM_000203.5:c.166del	n.d.	Not reported	2	This study	—
	NP_000194.2:p.Leu56fs					
	Exon 2					
49	NM_000203.5:c.222_226del	n.d.	n.d.	1	This study	—
	NP_000194.2:p.Leu74fs					
	Exon 2					
50	NM_000203.5:c.584_589+8del	n.d.	n.d.	1	This study	—
	Exon 5/intron6					
51	NM_000203.5:c.683del	CD169664	n.d.	1	Described in Korean patients with MPS I	Kwak et al. (2016)
	NP_000194.2:p.Pro228fs					
	Exon 6					
52	NM_000203.5:c.705_707del	n.d.	n.d.	1	This study	—
	NP_000194.2:p.Gly236del					
	Exon6					
53	NM_000203.5:c.923_932del	n.d.	n.d.	1	This study	—
	NP_000194.2:p.Leu308fs					
	Exon 7					
54	NM_000203.5:c.967_969del	n.d.	n.d.	1	This study	—
	NP_000194.2:p.Val323del					
	Exon 7					
55	NM_000203.5:c.1045_1047del	CD113571	VCV000557885.1 likely pathogenic	1	Described in a patient with MPS I	Bertola et al. (2011)
	NP_000194.2:p.Asp349del					
	Exon 8					
56	NM_000203.5:c.1238_1264del	n.d.	VCV000593572.1 uncertain significance	5	This study	—
	NP_000194.2:p.Asp413_Leu421del					
	Exon 9					
57	NM_000203.5:c.1400del	n.d.	Not reported	1	This study	—
	NP_000194.2:p.Pro467fs					
	Exon 9					
58	NM_000203.5:c.1451_1480del	n.d.	Not reported	1	This study	—
	NP_000194.2:p.Gly485_Val494del					
	Exon 10					
59	NM_000203.5:c.1614del	CD931013	VCV000167191.7 pathogenic	1	Described in patients with MPS I	Scott et al. (1993), Vazna et al. (2009)
	NP_000194.2:p.His539fs					
	Exon 14					
					The change creates a premature translational stop signal	
60	NM_000203.5:c.1847del	n.d.	n.d.	1	This study	—
	NP_000194.2:p.Gly616fs					
	Exon 14					
SMALL INSERTIONS						
61	NM_000203.5:c.811_816dup	n.d.	n.d.	1	This study	—
	NP_000194.2:p.Ser271_Ile272dup					
	Exon 7					
62	NM_000203.5:c.878_889dup	CI951941	VCV000550382.4 Pathogenic/Likely pathogenic	2	Described in patients with MPS I	Voskoboeva et al. (1998), Beesley et al. (2001), Venturi et al. (2002), Bertola et al. (2011)
	NP_000194.2:p.Thr293_Tyr296dup					
	Exon 7					
					The variant c.878_889dupCCCCCATTTAC results in the insertion of four amino acids to the IDUA protein (p.Thr293_Tyr296dup) but otherwise preserves the integrity of the reading frame	
63	NM_000203.5:c.1093dup	n.d.	n.d.	1	This study	—
	NP_000194.2:p.Leu365fs					
	Exon 8					
64	NM_000203.5:c.1742dup	n.d.	n.d.	1	This study	—
	NP_000194.2:p.Tyr581Ter					
	Exon 13					
65	NM_000203.5:c.1781dup	n.d.	n.d.	1	This study	—
	NP_000194.2:p.Thr594fs					
	Exon 13					
SMALL INSDEL						
66	NM_000203.5:c.510delinsAAGTTCCA	n.d.	Not reported	5	This study	—
	NP_000194.2:p.His171fs					
	Exon 5					
67	NM_000203.5:c.1099_1107delinsAGGTCAC	n.d.	Not reported	1	This study	—
	NP_000194.2:p.Ala367fs					
	Exon 8					
68	NM_000203.5:c.1873_1888delinACA	n.d.	n.d.	6	This study	—
	NP_000194.2:p.Tyr625fs					
	Exon 14					
SITE-SPLICING SUBSTITUTIONS						
69	NM_000203.5:c.972+2T>C	CS930838	Not reported	1	Described in patients with MPS I	Scott et al. (1993)
70	NM_000203.5:c.1403-1G>T	n.d.	VCV000652306.2 likely pathogenic	4	Described in patients with MPS I	
					The current evidence indicates that the variant is pathogenic, but additional data are needed to prove that conclusively.	Pollard et al. (2013)
71	NM_000203.5:c.1403–3C>G	n.d.	n.d.	1	This study	—
72	NM_000203.5:c.1524+1G>A	n.d.	VCV000940552.2 likely pathogenic	1	This study	—
					The sequence change affects a donor splice site in intron 10 of the IDUA gene. It is expected to disrupt RNA splicing and likely results in an absent or disrupted protein product. This variant has not been reported in the literature in individuals with IDUA-related conditions. The available evidence indicates that the variant is pathogenic, but additional data are needed to prove that conclusively	
73	NM_000203.5:c.1650+5G>A	CS022107	VCV000092634.3 pathogenic/likely pathogenic	5	Described in patients with MPS I	Venturi et al. (2002), Vazna et al. (2009), Bertola et al. (2011)
		CS113580				
					The change falls in intron 11 of the IDUA gene. It affects a nucleotide within the consensus splice site of the intron	
					Algorithms developed to predict the effect of sequence changes on RNA splicing suggest that this variant is not likely to affect RNA splicing, but this prediction has not been confirmed by published transcriptional studies	

A total of 409 mutant alleles were identified. The common mutation NM_000203.5:c.208C>T was prevalent in the patient cohort and represented 55.0% of the total number of patient alleles. The NM_000203.5:c.1205G>A variant, which is widespread throughout the world, was detected in only 12 patients (22 of 409 alleles) and accounted for 5.37% of mutant alleles. A similar pattern was observed for the previously described mutation NM_000203.5:c.1139A>G (21 of 409 alleles; 5.1%). The recurrent mutations (detected twice or more) were as follows: NM_000203.5:c.187C>T (14/409; 3.4%), NM_000203.5:c.1115A>G (9/409; 2.2%), NM_000203.5:c.1A>C (8/409; 1.9%), NM_000203.5:c.1898C>A (7/409; 1.7%), NM_000203.5:c.1873_1888delinsACA (6/410; 1.47%), NM_000203.5:c.510delinsAAGTTCCA (5/409; 1.2%), NM_000203.5:c.1238_1264del (5/409; 1.2%), NM_000203.5:c.1598C>T (5/409; 1.2%), NM_000203.5:c.1650+5G>A (5/409; 1.2%), NM_000203.5:c.1688A>C (5/409; 1.2%), NM_000203.5:c.1403-1G>T (4/409; 0.97%), NM_000203.5:c.1459T>C (3/409; 0.73%), NM_000203.5:c.46_57del (2/409; 0.48%), NM_000203.5:c.166del (2/409; 0.48%), NM_000203.5:c.223G>A (2/409; 0.48%), NM_000203.5:c.250G>A (2/409; 0.48%), NM_000203.5:c.878_889dup (2/409; 0.48%), NM_000203.5:c.1029C>A (2/409; 0.48%), and NM_000203.5:c.1882C>T (2/409; 0.48%). Fifty mutant alleles were unique, that is, occurring in only one individual (Table 1 and Table 2).

The novel mutations included two nonsense mutations, 11 missense mutations, 10 small deletions, four small insertions, three small delins, and two site-splicing substitutions. Five small deletions, two insertions, and all delins were mutations with frameshift. The estimation of frequencies and *in silico* analysis using the bioinformatics tools (Mutation taster, PolyPhen-2, SIFT, PROVEAN) was performed for the newly found mutations. Mutations were also classified according to the ACMG criteria. All novel mutations were considered to be pathogenic or likely pathogenic (Table 3).

**TABLE 3:** The annotation of the novel mutations of the *IDUA* gene.

*n*/n	Variant	Mutation type	Allele frequency in Database ExAC, 1000G, gnomAD.	Mutation taster	Polyphen-2	SIFT	PROVEAN	ACMG Criteria	Variant classification
1	NM_000203.5:c.140G>A	Nonsense	Found once in gnomAD in heterozygote state. Reference ID: rs1239326698 Global frequency A=0.0002	Deleterious stop codon in position 47	NA	NA	NA	PVS1; PM2; PM3; PM4; PP3	Pathogenic
	NP_000194.2:p.Trp47Ter								
2	NM_000203.5:c.1219C>T	Nonsense	Not found	Deleterious stop codon in position 407	NA	NA	NA	PVS1; PM2; PM; PP3	Pathogenic
	NP_000194.2:p.Gln407Ter								
3	NM_000203.5:c.531C>G	Missense	Found once in gnomAD and ExAC in heterozygote state Reference ID: rs769331894 TOTAL FREQIENCY G=0.000003985	Deleterious protein feature: 176–178 strand lost	Probably damaging	Tolerated	Deleterious	PM1; PM2; PP3	Likely pathogenic
	NP_000194.2:p.Phe177Leu								
4	NM_000203.5:c.1150A>G	Missense	Not found	Deleterious protein feature: no protein features affected	Probably damaging	Damaging	Deleterious	PM1;PM2; PP3	Likely pathogenic
	NP_000194.2:p.Lys384Asn								
5	NM_000203.5:c.1166C>A	Missense	Not found	Benign protein feature: 385–393 helix lost	Possibly damaging	Damaging	Deleterious	PM2; PM4; PP3	Likely pathogenic?
	NP_000194.2:p.Ala389Asp								
6	NM_000203.5:c.1321T>A	Missense	not found	deleterious protein feature: 435–442 strand lost	Probably damaging	Damaging	Deleterious	PM1;PM2; PP3	Likely pathogenic
	NP_000194.2:p.Tyr441Asn								
7	NM_000203.5:c.1459T>C	Missense	Not found	Deleterious protein feature: 483–489 helix lost	Probably damaging	Damaging	Deleterious	PM1, PM2; PM3; PM2; PP3	Likely pathogenic
	NP_000194.2:p.Trp487Arg								
8	NM_000203.5:c.1505G>C	Missense	Not found	? protein feature: 498–505 helix lost	Probably damaging	Tolerated	Neutral	PM2; PM3; PP3	Likely pathogenic?
	NP_000194.2:p.Arg502Pro								
9	NM_000203.5:c.1513C>G	Missense	Not found	Deleterious protein feature: 498–505 helix lost	Probably damaging	Damaging	Deleterious	PM1; PM2; PM3; PP3	Likely pathogenic
	NP_000194.2:p.Arg505Gly								
10	NM_000203.5:c.1600T>C	Missense	Not found	Deleterious protein feature: 529–541 strand	Probably damaging	Tolerated	Deleterious	PM1; PM2; PM3 PP3	Likely pathogenic
	NP_000194.2:p.Ser534Pro								
11	NM_000203.5:c.1622G>T	Missense	Not found	Deleterious 529-541 strand lost 541–541 DISULFID lost	Probably damaging	Damaging	Deleterious	PM1; PM2; PP3	Likely pathogenic
	NP_000194.2:p.Cys541Phe								
12	NM_000203.5:c.1664G>C	Missense	Not found	Deleterious protein feature: 552–560 strand lost	Probably damaging	Damaging	Deleterious	PM1; PM2; PP3	Likely pathogenic
	NP_000194.2:p.Arg555Pro								
13	NM_000203.5:c.1676T>C	Missense	Not found	Deleterious protein feature: 552-560 strand lost	Probably damaging	Damaging	Deleterious	PM1; PM2 PM3 PP3	Likely pathogenic
	NP_000194.2:p.Leu559Pro								
14	NM_000,203.5:c.166del	Frameshift deletion	Not found	Deleterious stop codon in position 107	NA	NA	NA	PVS1; PM2; PM4; PP3	Pathogenic
	NP_000194.2:p.Leu56fs								
15	NM_000203.5:c.222_226del	Frameshift deletion	Not found	Deleterious stop codon in position 129	NA	NA	NA	PVS1; PM2; PM4; PP3	Pathogenic
	NP_000194.2:p.Leu74fs								
16	NM_000203.5:c.584_589+8del	Deletion	Not found	Deleterious - deletion of more than 2AA Alteration within used splice site, likely to disturb normal splicing	NA	NA	NA	PVS1; PM2; PP3	Pathogenic
17	NM_000203.5:c.705_707del	Deletion	Not found	Deleterious deletion of 1 or 2 AA stop codon in position 653	NA	NA	NA	PM2; PM4; PP3	Pathogenic
	NP_000194.2:p.Gly236del								
18	NM_000203.5:c.923_932del NP_000194.2:p.Leu308fs	Frameshift deletion	Not found	Deleterious stop codon in position 313	NA	NA	NA	PVS1;PM2; PM4; PP3	Pathogenic
19	NM_000203.5:c.967_969del	Deletion	Not found	Deleterious deletion of 1 or 2 AA stop codon in position 653	NA	NA	NA	PM2; PM4; PP3	Pathogenic
	NP_000194.2:p.Val323del								
20	NM_000203.5:c.1238_1264del	Deletion	Not found	Deleterious deletion of more than 2 AA stop codon in position 645	NA	NA	NA	PM2; PM4; PP3	Pathogenic
	NP_000194.2:p.Asp413_Leu421del								
21	NM_000203.5:c.1400del	Frameshift deletion	Not found	Deleterious stop codon in position 233	NA	NA	NA	PVS1; PM2; PM4; PP3	Pathogenic
	NP_000194.2:p.Pro467fs								
22	NM_000203.5:c.1451_1480del	Small deletion	Not found	Deleterious deletion of more than 2 AA stop codon in position 644	NA	NA	NA	PM2; PM4: PP3	Pathogenic
	NP_000194.2:p.Gly485_Val494del								
23	NM_000203.5:c.1847del	Frameshift Deletion	Not found	Deleterious No stop codon within CDS 37 extra AA in CDS	NA	NA	NA	PVS1; PM2; PM4: PP3	Pathogenic
	NP_000194.2:p.Gly616fs								
24	NM_000203.5:c.811_816dup	Small insertion	Not found	Deleterious insertion of 1 or 2 AA stop codon in position 656	NA	NA	NA	PM2; PM4: PP3	Pathogenic
	NP_000194.2:p.Ser271_Ile272dup								
25	NM_000203.5:c.1093dup	Frameshift insertion	Not found	Deleterious stop codon in position 398	NA	NA	NA	PVS1;PM2; PM4: PP3	Pathogenic
	NP_000194.2:p.Leu365fs								
26	NM_000203.5:c.1742dup	Insertion	Not found	Deleterious stop codon in position 581	NA	NA	NA	PVS1;PM2; PM4: PP3	Pathogenic
	NP_000194.2:p.Tyr581Ter								
27	NM_000203.5:c.1781dup	Insertion	Not found	Deleterious original stop codon lost, results in prolonged protein 658	NA	NA	NA	PVS1;PM2; PM4: PP3	Pathogenic
	NP_000194.2:p.Thr594fs								
28	NM_000203.5:c.510delinsAAGTTCCA	Frameshift deletion/insertion	Not found	Deleterious stop codon in position 184	NA	NA	NA	PVS1;PM2; PM4: PP3	Pathogenic
	NP_000194.2:p.His171fs								
29	NM_000203.5:c.1099_1107delinsAGGTCAC	Frameshift deletion/insertion	Not found	Deleterious stop codon in position 397	NA	NA	NA	PVS1;PM2; PM4: PP3	Pathogenic
	NP_000194.2:p.Ala367fs								
30	NM_000203.5:c.1873_1888delinsACA	Frameshift deletion/insertion	Not found	Deleterious no stop codon within CDS (33 extra AA in CDS)	NA	NA	NA	PVS1;PM2; PM4: PP3	Pathogenic
	NP_000194.2:p.Tyr625fs								
31	NM_000203.5:c.1403-3C>G	Site-splicing substitutions	Not found	Deleterious effect acceptor weakened	NA	NA	NA	PM2; PM3	Pathogenic
32	NM_000203.5:c.1524+1G>A	Site-splicing substitutions	Not found	Alteration within used splice site, likely to disturb normal splicing	NA	NA	NA	PVS1;PM2; PM3	Pathogenic

### Genotype–Phenotype Correlation

Of 98.5% patients (203 of 206) had two *IDUA* variants identified. In three patients (1.5%), only one mutant allele was found. Ninety-three different genotypes were detected, with 74 genotypes being unique (35.9% of all patients). One hundred and fifty-seven patients were classified as having a severe phenotype and 49 as an attenuated (Table 1).

### Patients With a Severe Phenotype (MPS IH)

There were 59 individual genotypes represented in the 157 patients with a severe phenotype; 14 genotypes were recurrent and 45 genotypes were unique. The most common genotypes in the patients were NM_000203.5:c.[208C>T]; [208C>T] (68/157; 43.3%), NM_000203.5:c.[208C>T]; [1205G>A] (11/157; 7.0%), and NM_000203.5:c.[187C>T]; [187C>T] (6/157; 3.8%). These three nonsense variants defined the genotypes of 54.1% (85/157) of the patients. A total of 91.7% (144/157) patients with MPS IH were either homozygous or compound heterozygous for two “null” variants (e.g., nonsense variants, frameshifts, consensus splice site disruption, or initiator codon mutation). A total of 7.6% (12/157) of the patients were compound heterozygous for missense/nonsense variant or missense/frameshift variant. Only one patient (0.63%) was homozygous for missense variant (Table 4).

**TABLE 4:** Patients with MPS I with a severe phenotype (*n* = 157).

*n*/n	Genotype	Genotype feature	Number of patients	Mutation type
1	NM_000203.5:c.[208C>T]; [208C>T]	Recurrent	68	N/N
2	NM_000203.5:c.[208C>T]; [1205G>A]	Recurrent	11	N/N
3	NM_000203.5:c.[187C>T]; [187C>T]	Recurrent	6	N/N
4	NM_000203.5:c.[208C>T]; [1598C>T]	Recurrent	4	N/M
5	NM_000203.5:c.[208C>T]; [1238_1264del]	Recurrent	3	N/DEL
6	NM_000203.5:c.[208C>T]; [1898C>A]	Recurrent	3	N/N
7	NM_000203.5:c.[208C>T]; [c.1650+5g>a]	Recurrent	3	N/SS
8	NM_000203.5:c.[208C>T]; [1029C>A]	Recurrent	2	N/N
9	NM_000203.5:c.[1205G>A]; [1898C>A]	Recurrent	2	N/N
10	NM_000203.5:c.[208C>T]; [223G>A]	Recurrent	2	N/M
11	NM_000203.5:c.[208C>T]; [1688A>C]	Recurrent	2	N/M
12	NM_000203.5:c.[1238_1264del]; [1459T>C]	Recurrent	2	DEL/M
13	NM_000203.5:c.[1A>C]; [1A>C]	Recurrent	2	INC./INC.
14	NM_000203.5:c.[1A>C]; [510delinsAAGTTCCA]	Recurrent	2	INC./FS
15	NM_000203.5:c.[1205G>A]; [1205G>A]	Unique	1	N/N
16	NM_000203.5:c.[187C>T]; [1030C>G]	Unique	1	N/N
17	NM_000203.5:c.[1601C>A]; [1743C>G]	Unique	1	N/N
18	NM_000203.5:c.[1882C>T]; [1882C>T]	Unique	1	N/N
19	NM_000203.5:c.[1898C>T]; [1898C>T]	Unique	1	N/N
20-24	NM_000203.5:c.208C>T in combination with unique nonsense: NM_000203.5:c.123G>A	Unique	5 total	N/N
	NM_000203.5:c.140G>A			
	NM_000203.5:c.1219C>T			
	NM_000203.5:c.1855C>T			
	NM_000203.5:c.1861C>T			
25-32	NM_000203.5:c.208C>T in combination with unique small deletion: NM_000203.5:с.35_46del	Unique	8 total	N/DEL
	NM_000203.5:c.222_226del			
	NM_000203.5:c.683del			
	NM_000203.5:c.705_707del			
	NM_000203.5:c.923_932del			
	NM_000203.5:c.1045_1047del NM_000203.5:c.1614del			
	NM_000203.5:с.1847del			
32-36	NM_000203.5:c.208C>T in combination with unique small insertion:	Unique	4 total	N/INS
	NM_000203.5:c.816_817dup			
	NM_000203.5:с.1092_1093dup			
	NM_000203.5:c.1742_1743dup			
	NM_000203.5:c.1781dup			
37	NM_000203.5:c.[208C>T]; [1099_1107deinsAGGTCAC]	Unique	1	N/FS
38	NM_000203.5:c.[1205G>A]; [1873_1888delinsACA]	Unique	1	N/FS
39	NM_000203.5:c.[510delinsAAGTTCCA]; [510delinsAAGTTCCA]	Unique	1	FS/FS
40	NM_000203.5:c.[166del]; [166del]	Unique	1	FS/FS
41	NM_000203.5:c.[1A>C]; [208C>T]	Unique	1	INC./N
42	NM_000203.5:c.[1A>C]; [187C>T]	Unique	1	INC./N
43	NM_000203.5:c.[208C>T]; [584_589+8del]	Unique	1	N/SS
44-46	NM_000203.5:c.208C>T in combination with unique s.s.substitution: NM_000203.5:c.972+2T>C, NM_000203.5:c.1403–3C>G, NM_000203.5:c.1524+1G>A	Unique	3 total	N/SS
47	NM_000203.5:c.[1205G>A]; [1403-1G>T]	Unique	1	N/SS
48	NM_000203.5:c.[1650+5G>A]; [1650+5G>A]	Unique	1	SS/SS
49	NM_000203.5:c.[1403-1G>T]; [1403-1G>T]	Unique	1	SS/SS
50	NM_000203.5:c.[1403-1G>T]; [1450_1480del]	Unique	1	SS/DEL
51	NM_000203.5:c.[46_57del]; [1873_1888delinsACA]	Unique	1	DELSP/FS
52-56	NM_000203.5:c.208C>T in combination with unique missense: NM_000203.5:c.223G>C	Unique	5 total	N/M
	NM_000203.5:c.979G>C,			
	NM_000203.5:c.1150A>G,			
	NM_000203.5:c.1459T>C,			
	NM_000203.5:c.1513C>G			
57	NM_000203.5:c.[250G>C]; [250G>C]	Unique	1	M/M
58	NM_000203.5:c.[653C>T]; [1398del]	Unique	1	M/FS
59	NM_000203.5:c.[1205G>A]; [?]	Unique	1	N/?

N, nonsense mutation; M, missense mutation; FS, mutation with frame shift; DEL, deletion (with or without frame shift); INS**,** insertion (with or without frame shift); INC., mutation in initiation codon; DELSP, deletion in signal peptide area.

### Patients With an Attenuated Phenotype (MPS IH/S, MPS IS)

There were 34 individual genotypes represented in the 49 patients with an attenuated phenotype. Five genotypes were recurrent and 29 genotypes were unique. The most common genotypes in the patients were NM_000203.5:c.[208C>T]; [1139A>G] (9/49; 18.4%), NM_000203.5:c.[208C>T]; [c.1115A>G] (4/49; 8.2%), and NM_000203.5:c.[1139A>G]; [1205G>A] (3/49; 6.1%). A total of 71.4% of patients with an attenuated phenotype (35/49) were heterozygous for a “null”/missense variant and 14.2% (7/49) were either homozygous or compound heterozygous for two missense variants. Within that patient cohort, 91.8% (45/49) of the patients had at least one missense variant. The remaining four genotypes were NM_000203.5:c.[208C>T]; [878_889dup], NM_000203.5:c.[208C>T]; [1873_1888delinsACA], NM_000203.5:c.[1873_1888delinsACA]; [1873_1888delinsACA], and NM_000203.5:c.[208C>T]; [?] (Table 5).

**TABLE 5:** Patients with MPS I with an attenuated phenotype. (*n* = 49).

*n*/n	Genotype//feature	Genotype feature	Number of patients	Mutation type
1	NM_000203.5:c.[208C>T]; [1139A>G]	Recurrent	9	N/M
2	NM_000203.5:c.[208C>T]; [1115A>G]	Recurrent	4	N/M
3	NM_000203.5:c.[1139A>G]; [1205G>A]	Recurrent	3	M/N
4	NM_000203.5:c.[1139A>G]; [1139A>G]	Recurrent	2	M/M
5	NM_000203.5:c.[1115A>G]; [1115A>G]	Recurrent	2	M/M
6-20	NM_000203.5:c.208C>T in combination with unique missense: NM_000203.5:c.266G>A	Unique	15 total	N/M
	NM_000203.5:c.531C>G			
	NM_000203.5:c.589G>A			
	NM_000203.5:c.793G>C			
	NM_000203.5:c.826G>A			
	NM_000203.5:c.1037T>G			
	NM_000203.5:c.1166C>A			
	NM_000203.5:c.1321T>A			
	NM_000203.5:c.1475G>C			
	NM_000203.5:c.1505G>C			
	NM_000203.5:c.1600T>C			
	NM_000203.5:c.1622G>T			
	NM_000203.5:c.1664G>C			
	NM_000203.5:c.1688A>C			
	NM_000203.5:c.1898C>T			
21	NM_000203.5:c.[1205G>A]; [1688A>C]	Unique	1	N/M
22	NM_000203.5:c.[208C>T]; [878_889dup]	Unique	1	N/INS
23	NM_000203.5:c.[208C>T]; [1873_1888delinsACA]	Unique	1	N/FS
24	NM_000203.5:c.[208C>T]; [?]	Unique	1	N/?
25	NM_000203.5:c.[510delinsAAGTTCCA]; [1049A>G]	Unique	1	FS/M
26	NM_000203.5:c.[1139A>G]; [1873_1888delinsACA]	Unique	1	M/FS
27	NM_000203.5:c.[46_57del]; [1139A>G]	Unique	1	DELSP/M
28	NM_000203.5:c.[1139A>G]; [1676T>C]	Unique	1	M/M
29	NM_000203.5:c.[1115A>G]; [1688A>C]	Unique	1	M/M
30	NM_000203.5:c.[718C>G]; [ 1044C>G]	Unique	1	M/M
31	NM_000203.5:c.[878_889dup]; [1598C>T]	Unique	1	INS/M
32	NM_000203.5:c.[967_969del]; [1139A>G]	Unique	1	DEL/M
33	NM_000203.5:c.[1139A>G]; [?]	Unique	1	M/?
34	NM_000203.5:c.[1873_1888delinsACA]; [1873_1888delinsACA]	Unique	1	FS/FS

N, nonsense mutation; M, missense mutation; FS, mutation with frame shift; DEL, deletion; INS, insertion; DELSP, deletion in signal peptide area.

### Epidemiology

#### Slavic Russian Population

A group of 173 Russian patients of Slavic origin was formed. The information on nationality beyond the second generation was not available. The parents of 169 patients considered themselves to be Russians. Four marriages were mixed: Russian/Turkmen, Russian/Armenian, Russian/Korean, and Russian/Azerbaijani. Parents also found it difficult to specify the relocation of their ancestors. The parents of all patients denied consanguineous marriages. Рatients and their families lived in different regions of the RF (Table 1). The predominant pathogenic variant among Russian patients was NM_000203.5:c.208C>T with a frequency of 60.6%. Sixty-four (36.9%) patients were homozygous for NM_000203.5:c.208C>T and 84 (47.3%) were heterozygous. The frequency of NM_000203.5:c.1205G>A accounted only for 5.8% in Russian patients (20 of 346 alleles). The NM_000203.5:c.1139A>G mutation occurred with almost the same frequency (19/346; 5.4%). Recurrent mutation among Russian patients were NM_000203.5:c.1115A>G (9/346; 2.6%), NM_000203.5:c.1873_1888delinsACA (6/345; 1.7%), NM_000203.5:c.1898C>A (5/346; 1.4%), NM_000203.5:c.1598C>T (5/346; 1.4%), NM_000203.5:c.1238_1264del (5/346; 1.4%), NM_000203.5:c.1650+5G>A (5/346; 1.4%), NM_000203.5:c.1688A>C (4/346; 1.2%), NM_000203.5:c.1459T>C (3/346; 0.86%), NM_000203.5:c.223G>A (2/346; 0.57%), NM_000203.5:c.1029C>A (2/346; 0.57%), and NM_000203.5:c.878_889dup (2/346; 0.57%). A total of 129 patients were considered MPS IH and 44 were considered MPS IH/S or MPS IS (Table 1).

### Tatar Population

Among nine unrelated patients of Tatar ethnicity, NM_000203.5:c.208C>T also predominated and accounted for 55% mutant alleles (10 of 18). Three patients were homozygous for NM_000203.5:c.208C>T and four were heterozygous with genotypes: NM_000203.5:c.[208C>T]; [1688A>C], NM_000203.5:c.[208C>T]; [1037T>G], NM_000203.5:c.[208C>T]; [1166C>A], and NM_000203.5:c.[208C>T]; [1099_1107delinsAGGTCAC]. The genotype of remaining patient was NM_000203.5:c.[46_57del]; [1139A>G]. In one Tatar patient, only allele NM_000203.5:c.1139A>G was detected. Five patients had severe form of disease, and four had attenuated form (Table 1).

### Turkic Origin Patients

Uzbeks, Kyrghyz, and Altaians are indigenous peoples of Turkic origin living in Central Asia. On the basis of the assumption of a single common ancestor, we assigned these patients to a group of Turkic origin. In the group, NM_000203.5:c.187C>T mutation prevailed. Variant NM_000203.5:c.187C>T was found in homozygous state in six patients (five Uzbeks and one Altaian) and in heterozygous state, in one Uzbek and one Kyrgyz. The frequency of NM_000203.5:c.187C>T was 77.7%. Mutation NM_000203.5:c.187C>T was first described by the authors in their previous study and has not been reported by anyone else (Voskoboeva et al., 1998). All patients had Hurler phenotype (Table 1).

### Armenian Population

Neither alleles NM_000203.5:c.208C>T nor allele NM_000203.5:c.1205G>A were found in six unrelated Armenian patients. The determined pathogenic alleles were NM_000203.5:c.510delinsAAGTTCCA (5 of 12 alleles), NM_000203.5:c.1A>C (4/12), NM_000203.5:c.1898C>A (2/12), NM_000203.5:c.1049A>G (1/12). All but one (#192) patients had the severe phenotype (Table 1).

### Kazakh Population

In three unrelated Kazakh patients, the prevalent mutation was NM_000203.5:c.1403-1G>T (four of the six alleles). The remaining alleles were NM_000203.5:c.1205G>A and a novel minor deletion NM_000203.5:c.1451_1480del. All patients had severe form of disease (Table 1).

### Azeri Population

Two patients with a severe phenotype of Azerbaijani nationality were homozygous for missense variants: NM_000203.5:c.1A>C and NM_000203.5:c.250G>C (Table 1).

### Ukrainian Patients

In two Ukrainian patients with MPS IH, the NM_000203.5:c.208C>T allele was found in homozygous state and in combination with site-splicing substitution NM_000203.5:c.972+2T>C (Table 1).

## Discussion

### DNA Analysis and Epidemiology

The Soviet Union was a state in Eurasia that existed from 1922 to 1991. In addition to the Russian Republic, there were 14 other republics, each with its own national composition. Representatives of more than 200 different nationalities (ethnic groups) live in today’s Russia. About 80% of the population of Russia are Russians. There were no representatives of ethnic groups from Russian regions among the examined Russian patients, with the exception of one Altaian (Altai Republic) and one Avar (Dagestan Republic). Thus, the group of Russian patients was represented by Russians of Slavic origin. Patients’ families lived in different regions of the country. Unfortunately, there was no information on possible resettlement of the families. To simplify the analysis, we divided patients’ places of residence according to the federal districts of the RF (Table 1).

The Tatars are the second largest nation in the RF after the Russians. Mutation NM_000203.5:c.208C>T was found to be predominant among Russian and Tatar patients. Two siblings and 62 unrelated Russian patients and three unrelated Tatar patients were homozygous for NM_000203.5:c.208C>T. Eighty-four unrelated Russian and four Tatar patients were heterozygous for NM_000203.5:c.208C>T.

**FIGURE 1:**
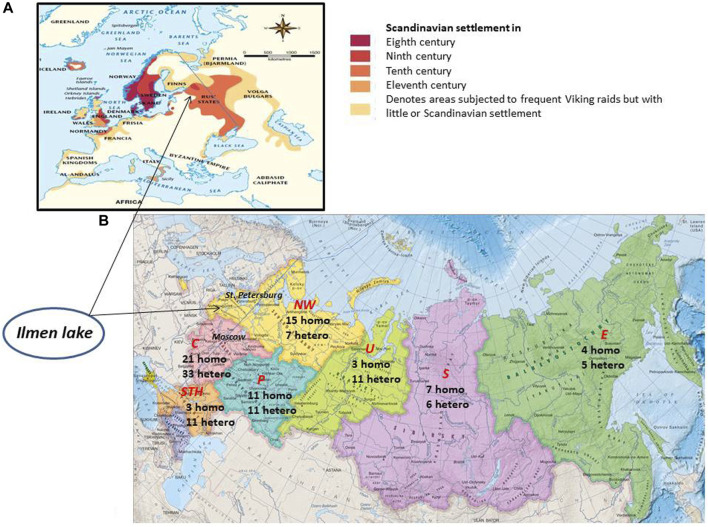
**(A)** Alleged settlement of the Scandinavians in ancient times. **(B)** Distribution of NM_000203.5:c.208C>T among Russian patients living in different regions of the Russian Federation. Federal districts of the Russian Federation are highlighted in red italics. C, central; NW, Northwest; STH, South; P, Privolzhsky; U, Ural; S, Siberia; E, Far East. The digits indicate the number of patients; homo, homozygote for NM_000203.5:c.208C>T; hetero, heterozygote for NM_000203.5:c.208C>T.

The NM_000203.5:c.208C>T is one of the most common pathogenic variants in the IDUA gene, accounting for up to 19%–62% of pathogenic alleles among North and East European or Scandinavian patients with MPS I. The frequency of NM_000203.5:c.208C>T decreases from the north to the south across Europe (Khan et al., 2017; Poletto et al., 2018). Such distribution of NM_000203.5:c.208C>T is explained by the possible Viking origin of the allele (Poletto et al., 2018). It is assumed that, in the eighth century, the *Scandinavian colonial expansion* began, moving mainly along the coast of the Baltic and North Seas. The Vikings also migrated eastward across the territories of the present-day Russia. At the same time, the eastern Slavs inhabited a large part of the East European plain, reaching the Lake Ilmen in the north. According to the current hypothesis, the historical settlements of the Scandinavians may have looked as follows (Figure 1A).

The hypothesis of NM_000203.5:c.208C>T origin is consistent with the observed pattern of allele accumulation in the Central, Northwestern, and Volga territories of modern Russia, with decreasing frequency in Siberia and the Far East (Figure 1B). It is possible that the high accumulation of NM_000203.5:c.208C>T homozygotes is explained by the founder effect, and the historical migration of the population to Siberia and the East has led to a dilution of the prevalence of homozygotes. Similar data were obtained in our first study (Voskoboeva et al., 1998). Tatar patients were few, so the frequency of NM_000203.5:c.208C>T may be overestimated. However, the accumulation of NM_000203.5:c.208C>T in Tatar patients could also be attributed to descent from a common ancestor.

Оn the other hand, Vazna A et al. showed that mutation NM_000203.5:c.208C>T might have arisen more than once (Vazna et al., 2009). Thus, it could be assumed that NM_000203.5:c.208C>T has a different origin in the population of Russians and, especially, Tatars.

In contrast to NM_000203.5:c.208C>T, the common allele NM_000203.5:c.1205G>A found with a high frequency among various populations in Europe, North America, and Australia was identified in only 11 Russian patients and only once in the homozygous state (Clarke and Scott, 1993; Poletto et al., 2018). A very similar pattern was observed for the NM_000203.5:c.1139A>G allele. The frequencies of these mutations did not exceed 5%. The variant NM_000203.5:c.1139A>G has been described in several patients of European origin and was predominantly (10%) encountered in patients with MPS I from the Czech Republic and Slovakia (Scott et al., 1995; Venturi et al., 2002; Matte et al., 2003; Vazna et al., 2009). Such a low frequency of these mutations is probably due to the insignificant resettlement of the European population from the west, which led to allele dilution in the Russian population. Allele NM_000203.5:c.1115A>G was the fourth most common in the Russian population (2.6%). The mutation NM_000203.5:c.1115A>G has been detected in Ukrainian patients and a patient from India (Trofimova, 2016; Uttarilli et al., 2016). There have been no reports of this mutation in other populations.

Turkic peoples are diverse ethnic groups defined by Turkic languages. According to a recent study, Kyrgyz, Kazakhs, Uzbeks, and Turkmens share more of a gene pool with various East Asian and Siberian populations than with West Asian or European populations (Yunusbayev et al., 2015). Another study suggests that Mongolian expansion has left a strong mark on the gene pool of Turkic peoples (Zerjal et al., 2002). The presence of a common ancient ancestor for certain Turkic-speaking groups could not be excluded. Variant NM_000203.5:c.187C>T might be arisen from a common ancestor and be a founder mutation for patients of Turkic origin.

Specific mutation pattern was found in the patients of the Armenian and Kazakh populations. Although only few patients were diagnosed, some features can be noted: 1. the absence of common alleles NM_000203.5:c.208C>T and NM_000203.5:c.1205G>A in patients in of these population groups; 2. recurrence of NM_000203.5:c.1A>C mutation among Armenians; 3. the prevalence of NM_000203.5:c.510delinsAAGTTCCA among Armenians and NM_000203.5:c.1403-1G>T, among Kazakhs. These findings are in agreement with the data on the specificity of the genetic background of MPS I in each population (Lee et al., 2004; Wang et al., 2012; Atçeken et al., 2016; Poletto et al., 2018). Mutation NM_000203.5:c.1A>C has been reported in Turkish, Chinese, and Spanish population (Bertola et al., 2011; Wang et al., 2012; Shafaat et al., 2019) and was most common in Iranian patients (Atçeken et al., 2016). The nucleotide variant NM_000203.5:c.1403-1G>T was described only in Chinese patients with MPS I (Pollard et al., 2013).

A recurrent mutation, especially in the homozygous state, can be caused by consanguinity. In unrelated families, a recurrent mutation can be a “hot spot” or founder mutation. The pattern of distribution of mutant alleles worldwide suggests that the accumulation of IDUA mutations is probably due to the founder effect. Although this is most likely true for NM_000203.5:c.208C>T in Russians and possibly in Tatars, the question remains open for mutations found in other populations. We can assume, on the basis of the different places of residence, that the patients were not related. However, this information was not obtained from all parents. Therefore, there is a possibility that the frequencies of homozygotes are associated with consanguineous marriages.

### Genotype–Phenotype Correlation

Because the material was collected over a long period of time, it was problematic in many cases to obtain detailed information about on patients’ phenotypes. Therefore, the analysis of genotype–phenotype correlation was performed in a reductive manner, as has been done by Clarke et al. (Clarke et al., 2019). Two groups of patients were formed: patients with a severe phenotype (MPS IH) and patients with an attenuated phenotype (MPS IH/S), with the exception of a few patients who were exactly classified as MPS IS (Table 1). In general, our data are in agreement with the data presented by the others (Venturi et al., 2002; Vazna et al., 2009; Bertola et al., 2011; Prommajan et al., 2011; Clarke et al., 2019). All patients homozygous for two “null” alleles had Hurler phenotype. Most patients with an attenuated phenotype had at least one allele represented by a missense mutation. Phenotype divergence was observed in patients with NM_000203.5:c.[208C>T]; [1688A>C] genotype (#135, #136, and #172).

Patients heterozygous for NM_000203.5:c.878_889dup in combination with NM_000203.5:c.208C>T and NM_000203.5:c.1598C>T (#152 and #105) had an attenuated form of the disease. Moreover, patient #105 had an extremely mild form of MPS I. She is now 42 years old and has given birth to two children. Professionally, she has a degree in geography. The patient was first described in our study 23 years ago (Voskoboeva et al., 1998). At the same time, patients’ genetic compounds NM_000203.5:c.[208C>T]; [1598C>T] (## 101-104) were classified as MPS IH (Tables 1, 4, 5). Consistent with other authors (Vazna et al., 2009), we found mutation NM_000203.5:c.1139A>G in two siblings with the Scheie phenotype (#91 and #91a) and in combination with NM_000203.5:c.208C>T or NM_000203.5:c.1205G>A in patients with MPS IH/S or MPS IS (#76 to #78 and #82 to #90). Patients with genotypes NM_000203.5:c.[1139A>G]; [1676T>C] (#92), NM_000203.5:c.[967_969del]; [1139A>G] (#93), and NM_000203.5:c.[1139A>G]; [1873_1888delinsACA (#94) also had an attenuated form of disease (Table 1).

Вoth groups of researchers who described the NM_000203.5:c.1115A>G mutation reported it in patients with a severe phenotype (Trofimova, 2016; Uttarilli et al., 2016). Trofimova et al. suggested that NM_000203.5:c.1115A>G substitution leads to a change in the splice site, but there are no data on the functional study performed. Three our patients heterozygous for NM_000203.5:c.1115A>G had the attenuated form of the disease (#95 to #97). Other two heterozygous patients (#98 and #100) and two homozygous siblings (#99 and #99a) were classified as MPS IS (Table 1).

We were able to identify the genetic features of MPS I among the patients of such a multipopulation country as the Former Soviet Union. Knowledge of MPS I genetic background in each population is very important for providing patients with the right care. Determination of prevalent mutations will allow creating cost-effective test systems and avoiding unnecessary testing for a multitude of rare variants. It may also help in developing national screening programs or designing new genotype-specific treatments.

To highlight some of the findings, our data show the following: 1. the standard approach to the *IDUA* gene DNA analysis identified 98.5% of the genotypes; 2. an accumulation of the NM_000203.5:c.208C>T mutation among Russian patients was detected, which is probably attributed to the founder effect. The frequency of NM_000203.5:c.208C>T is very close to that in Scandinavian countries, which may reflect the existing hypothesis of a Viking origin of NM_000203.5:c.208C>T; 3. common NM_000203.5:c.208C>T and NM_000203.5:c.1205G>A alleles were rare or absent among patients from other ethnic groups (except Tatars and Ukrainians). The prevalence of their unique alleles was detected among these patients. These results are in agreement with those of other researchers; 4. the analysis of genotype–phenotype correlations did not reveal any principal discrepancies with the conclusions of other researchers. A significant discrepancy occurred only for the NM_000203.5:c.1115A>G.

This study also has a number of limitations: 1. 76.2% of the patients in the cohort had a severe phenotype and thus clearly marked clinical manifestations. It could not be excluded that patients with an attenuated form of the disease remain underdiagnosed; 2. at least one study reported a possible non-single origin of NM_000203.5:c.208C>T, which calls into question the founder mutation effect associated with Viking ancestry; 3. in many cases, data on clinical phenotypes were poor and, often, determined by the subjective opinion of the physician, making it difficult to perform genotype–phenotype correlation analysis; 4. the frequencies of unique alleles in the populations examined may be overestimated because of few patients diagnosed; 5. analysis of novel mutations was performed only *in silico*; 6. not all patients’ parents’ DNA was available for testing.

A more careful analysis of the patient history, possibly based on certain clinical criteria, is needed to allow the physician to distinguish between MPS IH, MPS IH/S, and MPS IS. A functional analysis for detectable mutations in the *IDUA* gene, especially missense variants, is required to evaluate their actual effect on enzyme function. Parental DNA testing is necessary to confirm inheritance of the disease. When recurrent mutation is observed in unrelated patients, a detailed analysis of polymorphic the *IDUA* gene variants and haplotypes is needed to distinguish the “hot spot” from the founder mutation.

## Data Availability

The original contributions presented in the study are included in the article/Supplementary Material, further inquiries can be directed to the corresponding authors.
